# Signature of quantum Griffiths singularity state in a layered quasi-one-dimensional superconductor

**DOI:** 10.1038/s41467-018-07123-y

**Published:** 2018-11-07

**Authors:** Enze Zhang, Jinhua Zhi, Yi-Chao Zou, Zefang Ye, Linfeng Ai, Jiacheng Shi, Ce Huang, Shanshan Liu, Zehao Lin, Xinyuan Zheng, Ning Kang, Hongqi Xu, Wei Wang, Liang He, Jin Zou, Jinyu Liu, Zhiqiang Mao, Faxian Xiu

**Affiliations:** 1 State Key Laboratory of Surface Physics and Department of Physics, Fudan University, 200433 Shanghai, China; 2 Institute for Nanoelectronic Devices and Quantum Computing, Fudan University, 200433 Shanghai, China; 3 Bejing Key Laboratory of Quantum Devices, Key Laboratory for the Physics and Chemistry of Nanodevices and Department of Electronics, Peking University, 100871 Beijing, China; 4 Materials Engineering, The University of Queensland, Brisbane, QLD 4072 Australia; 5 Centre for Microscopy and Microanalysis, The University of Queensland, Brisbane, QLD 4072 Australia; 6 School of Electronics Science and Engineering, Nanjing University, 210093 Nanjing, China; 7 Department of Physics and Engineering Physics, Tulane University, New Orleans, LA 70118 USA; 8 Collaborative Innovation Center of Advanced Microstructures, Nanjing University, 210093 Nanjing, China

## Abstract

Quantum Griffiths singularity was theoretically proposed to interpret the phenomenon of divergent dynamical exponent in quantum phase transitions. It has been discovered experimentally in three-dimensional (3D) magnetic metal systems and two-dimensional (2D) superconductors. But, whether this state exists in lower dimensional systems remains elusive. Here, we report the signature of quantum Griffiths singularity state in quasi-one-dimensional (1D) Ta_2_PdS_5_ nanowires. The superconducting critical field shows a strong anisotropic behavior and a violation of the Pauli limit in a parallel magnetic field configuration. Current-voltage measurements exhibit hysteresis loops and a series of multiple voltage steps in transition to the normal state, indicating a quasi-1D nature of the superconductivity. Surprisingly, the nanowire undergoes a superconductor-metal transition when the magnetic field increases. Upon approaching the zero-temperature quantum critical point, the system uncovers the signature of the quantum Griffiths singularity state arising from enhanced quenched disorders, where the dynamical critical exponent becomes diverging rather than being constant.

## Abstract (web summary)

Whether the quantum Griffiths singularity state exists in one-dimensional (1D) systems remains elusive. Here, Zhang et al. report violation of the Pauli limit in the superconducting critical field and multiple phase transitions in the current-voltage hysteresis loops in a Ta_2_PdS_5_ nanowire, suggesting signatures of quasi-1D quantum Griffith singularity.

## Introduction

Superconductivity in materials with low-dimensional electronic structure becomes an important topic during the past decades because they provide a rich avenue for investigating exotic physical properties and their potential applications in quantum computing devices^1,2^. With the reduction of the dimensionality, fluctuation, disorder, and quantum correlation effects begin to have a special influence on the superconducting characteristics^3^. As a result, many interesting phenomena arise, such as localization of Cooper pairs^4^, transition temperature oscillations^5,6^, and quantum phase transition (QPT) at zero temperature^7–9^. In particular, one example of the QPT has been extensively studied that is the superconductor-insulator transition (SIT) or superconducting-metal transition (SMT), in which a continuous phase transition occurs at the zero-temperature limit as a function of external tuning parameters, such as external electric fields, carrier density, and out-of-plane magnetic fields^1,9^. However, despite numbers of investigations in various systems, there still remain many open issues, such as unexplained inconsistencies among the critical exponents found in different physical systems^1,10^ and the appearance of intervening quantum metallic state between the superconducting and the insulating state^2,11^. The latest remarkable observations of the quantum Griffiths singularity in thin Ga films^12^, LaAlO_3_/SrTiO_3_(110) interface^13^, monolayer NbSe_2_^14^, and ionic liquid gated ZrNCl and MoS_2_^15^ shed a new light on the understanding of SMT in two-dimensional (2D) system^16^. Whether this phenomenon would appear in lower dimensional superconducting systems remains elusive.

Very recently, the newly discovered quasi-one-dimensional (1D) transition metal chalcogenide superconductors with a formula M_2_Pd_x_Q_5_ (M = Ta, Nb; Q = S, Se) have attracted great attention because of their exotic superconducting characteristics^17,18^. The most striking feature of these compounds is their remarkably large upper critical fields, which exceed the Pauli limit relative to their transition temperature^19,20^. Theoretical investigations show that this should be due to the combination of strong spin-orbit coupling and multiband effects in the extreme dirty limit^21^. More interestingly, instead of using the sputtering or evaporating technique in fabricating granular and amorphous nanowires, the layered low-dimensional nature in such compounds offers a wonderful platform for investigating SMT in quasi-1D single-crystal nanowires^17^.

In this study, we report the systematic transport measurements on quasi-1D Ta_2_PdS_5_ nanowires obtained by Scotch tape assisted micromechanical exfoliation of bulk crystals. The critical field of the system shows a strong anisotropic behavior and a violation of Pauli paramagnetic law under parallel magnetic field configuration. Moreover, the current-voltage (*I-V*) characteristics of the nanowires in the superconducting regime display a series of multiple voltage steps resulting from quantum phase slip; and a hysteresis arises when the applied current is swept up and down, which resembles a typical quasi-1D behavior in superconductivity. Interestingly, the magnetotransport properties of the nanowires undergo a SMT with increasing magnetic field. As the temperature approaches zero, the dynamical critical exponent shows a divergent value, which is a signature of quantum Griffiths singularity state.

## Results

### Sample characterization

**Fig. 1:**
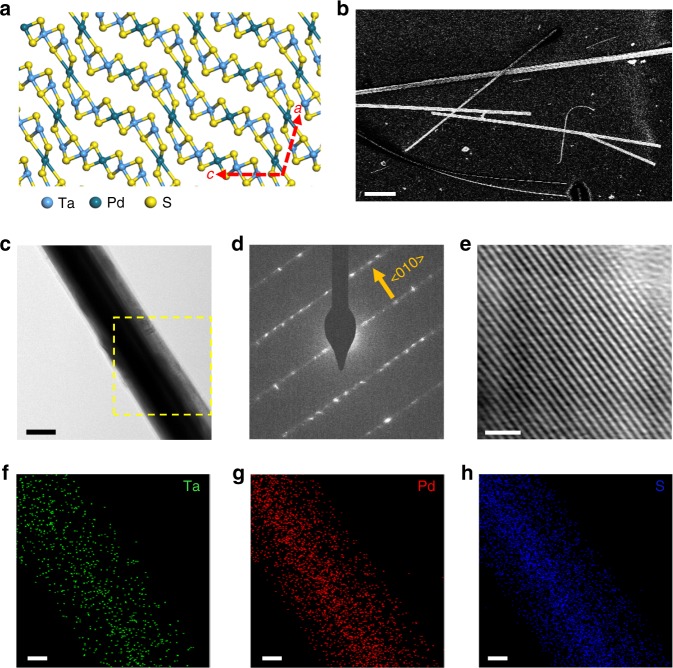
Crystal structure and characterizations of Ta_2_PdS_5_ crystals. **a** Crystal structure of Ta_2_PdS_5_ viewed along the *b*-axis, exhibiting layered structure in the plane perpendicular to *a*-axis of the crystal. **b** SEM image of the exfoliated Ta_2_PdS_5_ nanowire. Scale bar, 5 μm. **c** Bright-field TEM image taken from a section of a typical Ta_2_PdS_5_ nanowire. Scale bar, 100 nm. **d** Corresponding SAED pattern showing that the axial growth direction of the nanowire is <010> . **e** Corresponding HRTEM. Scale bar, 100 nm. **f**–**h** EDS elemental maps taken from the region marked by a square in **c**, for Ta (**f**), Pd (**g**), and S (**h**). Scale bars, 50 nm

Ta_2_PdS_5_ single crystals were synthesized using chemical vapor transport (CVT) method^22^ (see Methods for details). The as-grown bulk crystals exhibit a long needle-like structure with a length up to centimeters. As shown in Fig. 1a, Ta_2_PdS_5_ is composed of corrugated metal sulfide sheets, which form 1D chains along the < 010 > direction (needle direction or *b*-axis)^19,21^. Its crystal structure is centrosymmetric, with the space group of C2/m. Due to its low-dimensional structure, the Ta_2_PdS_5_ bulk material can be cleaved into rectangular-shape nanowires where the width ranges from 0.1 to 2 μm, with the thickness of 70–300 nm. Figure 1b shows the scanning electron microscope (SEM) image of the exfoliated nanowires on polydimethylsiloxane substrate, where the long axis of the nanowire is the *b*-axis of the crystal. Figure 1c shows the bright-field transmission electron microscopy (TEM) image taken from a Ta_2_PdS_5_ nanowire. Figures 1d, e respectively show the corresponding selective-area electron diffraction (SAED) pattern and high-resolution TEM (HRTEM) image, which confirm that the axial direction of the Ta_2_PdS_5_ nanowire is < 010 > and the single-crystal nature of the nanowire. Figures 1f–h show the elemental maps taken from a section of the nanowire (yellow dashed square in Fig. 1c) by energy-dispersive X-ray spectroscopy (EDS), indicating that Ta, Pd, and S are distributed evenly in the nanowire.

### Anisotropic superconductivity in Ta_2_PdS_5_ nanowires

**Fig. 2:**
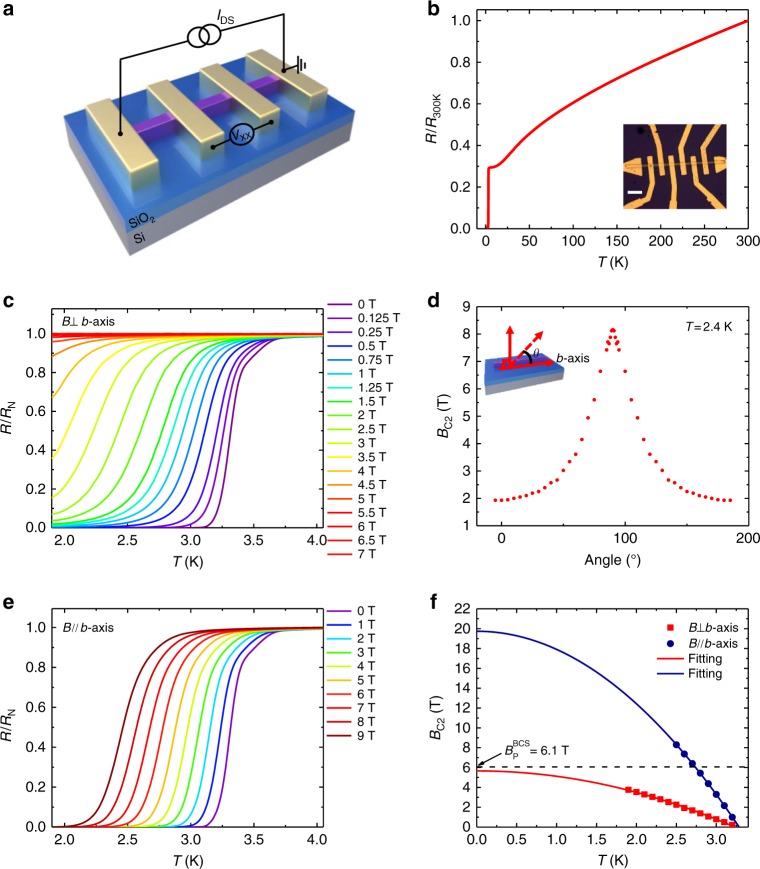
Anisotropic superconducting behavior in Ta_2_PdS_5_ nanowire device. **a** Schematic device structure based on a Ta_2_PdS_5_ nanowire. **b** Temperature dependence of the normalized resistance (red solid line) of Ta_2_PdS_5_ nanowire device with a cross-sectional area of 120 nm (thickness) × 300 nm (width). Inset, an optical image of the Ta_2_PdS_5_ device, scale bar, 5 μm. **c** The temperature-dependent resistance of the device under a magnetic field perpendicular to the substrate, the resistance is normalized to the normal-state value right above the superconducting transition. **d** Angular dependence of the critical magnetic field *B*_C2_ of a similar device measured at 2.4 K. Inset, Schematic configuration of angular-dependent magnetoresistance measurement. The *θ* = 0 is defined as the magnetic field parallel to the *b*-axis. **e** Normalized *R-T* characteristics of Ta_2_PdS_5_ under the magnetic field parallel to the *b*-axis. **f** Temperature-dependent *B*_C2_ for the magnetic field parallel and perpendicular to the *b*-axis. The red and blue solid lines are the theoretical fitting of *B*_C2_(*T*) *=* *B*_C2_(0)[1−(*T*/*T*_C_)^2^]. The black dashed line is the Pauli limit in response to the superconducting transition temperature

Four-terminal Ta_2_PdS_5_ nanowire devices with various thickness were fabricated using *e*-beam lithography (EBL), followed by a metal deposition process (See Methods for details). A typical device structure is schematically illustrated in Fig. 2a (Optical image, inset of Fig. 2b). The measured four-probe resistance is defined as *R* *=* *V*_XX_*/I*_DS_, where *I*_DS_ is the source-drain current and *V*_XX_ is the measured voltage drop between the middle two voltage probes. As depicted in Fig. 2b, four-terminal temperature-dependent resistance of the nanowire device shows a metallic behavior upon cooling and the superconductivity appears at *T*_C_ ∼ 3.3 K (Device 01 cross-sectional area, 120 nm (thickness) × 300 nm (width)), where *T*_C_ is defined as the temperature corresponding to the middle point of the superconducting transition. *T*_C_ values of devices with different thickness are shown in Supplementary Figure 3 in which *T*_C_ decreases monotonically with the thickness. Note that in order to control the variables, we intentionally compare the samples with a similar channel width. We also measure the *T*_C_ of different lengths in one nanowire (Supplementary Figure 3) and different parts of the nanowire exhibit a similar *T*_C_, indicative of good uniformity^17^.

Next, we explore the anisotropy of the superconductivity in Ta_2_PdS_5_. Figures 2c, e display the temperature-dependent resistance for magnetic fields applied perpendicular and parallel to the *b*-axis of Ta_2_PdS_5_ nanowire device (inset Fig. 2d), respectively. In the case of the perpendicular geometry, the superconductivity rapidly disappears with the increase of magnetic field and is completely quenched when the magnetic field is larger than 6.5 T. For the parallel scenario, however, the superconductivity is robust against the magnetic field and it survives even under 9 T at *T* = 2 K. Figure 2d shows the angle-dependent upper critical field *B*_C2_(*T*) at *T* = 2.4 K (similar device to device 01, the data of device 01 are shown in Supplementary Figure 4), where it exhibits large anisotropy. We define *B*_C2_(*T*) as the magnetic field where the resistance drops to 50% of the normal resistance. We tried to fit the angular-dependent *B*_C2_(*T*) at small *θ* values using both 3D^23,24^ and 2D^25,26^ models, where neither of them fits the data well (See Supplementary Figure 6). Then, we further measured the angle-dependent *B*_C2_(*T*) of Ta_2_PdS_5_ samples with different thickness (Supplementary Figure 7). We find that the angle-dependent *B*_C2_ ~5 μm (Bulk) sample shows a very good fit to the 3D model, which is consistent with the previous study^27^. However, the angle-dependent *B*_C2_ of thin nanowire samples do not fit well with either 3D or 2D model, indicating the reduced dimensionality of its superconductivity (See Supplementary Note 2 for details). As far as we know, up to now, there is no theoretical models/equations that can be used for the data fitting in the quasi-1D superconducting system, which calls for a further theoretical investigation. In addition, the dependence of *B*_C2_ on temperature *T* for both the parallel and perpendicular magnetic fields are fitted well using the empirical equation^17^:

1 $$B_{\mathrm{C2}}\left( T \right) = B_{\mathrm{C2}}\left( 0 \right)\left( 1 - \frac{T}{T_{\mathrm{C}}} \right)^2,$$

where *B*_C2_(0) = *Φ*_0_/2*πξ*(0)^2^. Then, the Ginsburg–Landau (GL) coherence length for parallel and perpendicular configuration can be estimated to be *ξ*_//_(0) = 4.1 nm and *ξ*_⊥_(0) = 7.6 nm, respectively. Considering the relatively small anisotropy in the *a-c* plane (See Supplementary Note 3 for details), the coherence along *a-* and *c*-axis should be around 4.1 nm. Note that for the parallel configuration, the upper critical field at *T* = 0 K seemingly exceeds the Pauli paramagnetic limit of weak coupling Bardeen–Cooper–Schrieffer (BCS) superconductors *B*_P_^BCS ^= *Δ*_0_/$\sqrt 2$*k*_B_*T*_C_ = 1.84*T*_C _= 6.1 T (dashed line in Fig. 2f). Compare with spin-momentum lock induced Ising superconductivity in monolayer NbSe_2_^14,28^ and ionic liquid gated MoS_2_^26^, the violation of Pauli paramagnetic limit in Ta_2_PdS_5_ is resulted from the synergetic effect of strong spin-orbit coupling and multiband effects^21,29^.

### *I-V* characteristics of Ta_2_PdS_5_ nanowire devices and the estimation of penetration depth

**Fig. 3:**
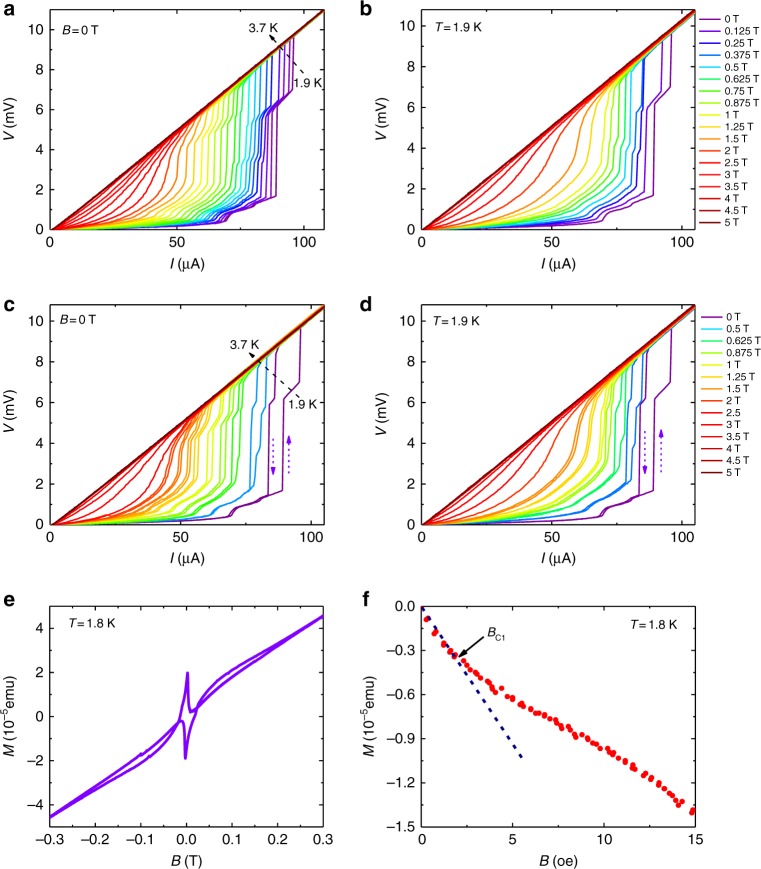
*I-V* characteristics and magnetic properties of Ta_2_PdS_5_. **a** Temperature-dependent *I-V* curves of the Ta_2_PdS_5_ nanowire in the linear scale under increased current bias, showing multiple voltage steps in the superconducting transition regime. **b** *I-V* curves of the Ta_2_PdS_5_ nanowire under various magnetic fields perpendicular to the substrate, in which multiple voltage steps were also observed under various magnetic fields. **c**, **d** Hysteresis loops in *I-V* curves when the current sweeps up and down at different temperatures and perpendicular magnetic fields, respectively. **e** Magnetic moment as a function of magnetic field in Ta_2_PdS_5_, which shows a typical behavior of type-II superconductor. **f** Expanded region of the initial magnetization curve up to 15 Oe in Ta_2_PdS_5_, where *B*_C1_(1.8 K) = 1.8 Oe can be extracted

In order to further understand the superconductivity in Ta_2_PdS_5_ nanowires, we performed four-terminal current-voltage (*I-V*) measurements in the superconducting transition region. Figure 3 shows the measured *I-V* curves at varying temperatures (Fig. 3a) and magnetic fields (Fig. 3b) on a linear scale driven by current. A series of sharp voltage steps were observed as the nanowires transit from the superconducting to the normal state. These voltage steps are reproducible, becoming more pronounced and sharper at lower temperatures (magnetic fields) and finally vanished with the gradual increase of temperature (magnetic field). Usually, the multiple voltage steps in the *I-V* characteristics are considered to be typical features of quasi-1D superconductors. It has been reported in thin superconducting polycrystalline Nb nanowires^30,31^ and single-crystal Sn nanowires^32^. These voltage steps were interpreted as a consequence of spatially localized weak spots or resistive phase slip centers (PSCs), which arise from the local imperfections or defects in the nanowire that support a smaller critical current. A voltage step in the wire is created when the applied excitation current exceeds the local critical current of a specific PSC. The spatial extension of the PSC is typically on the order of a few micrometers in length^32^, which is at the same level of our device length. In addition, when we sweep the current upstream and back down, a sequence of hysteresis loop has been observed in the superconducting regime (Figs. 3c, d), which also disappears as the temperature or magnetic field increases. As the phase-slip centers in superconducting nanowires act qualitatively like weak-link in Josephson junctions, a superconducting nanowire can be viewed as the coupled combination of Josephson junctions and the rest of the superconducting filament^33^. Hence, such a hysteresis in *I-V* characteristics is a hybrid effect resulting from both self-heating hotspots and the runaway and retrapping of the phase point in the tilted washboard-like potential of the underdamped Josephson junction^34^. We have also compared the *I-V* relation of Ta_2_PdS_5_ nanowires with thickness changes from ~5 μm (bulk) to 110 nm (See Supplementary Figure 9). For ~5 μm thick (Bulk) sample, there is only one step in the *I-V* curves and the voltage is zero under low current bias until the current reaches a certain value *I*_C_ (Note that the data points in Supplementary Figure 9(a) at *V*~1 μV are the noise approaching the measurement limit). As the thickness of the device goes down, multiple voltage steps and enhanced Ohmic finite resistance emerge, which is mainly due to the 1D confinement effect as predicted by thermal-activated phase slip model^32,35,36^ (See Supplementary Note 4 for details). In addition, we have fit the temperature-dependent critical current to the Bardeen’s formula^37,38^ for quasi-1D superconductors *I*(*T*) = *I*_*C*_(0) (1−(*T*/*T*_C0_)^2^)^3/2^, where *T*_C0_ is the transition temperature *T* in the absence of currents and fields (See Supplementary Figure 10a for details). We found that the experimental data fit the equation well, evidencing the quasi-1D superconductivity in Ta_2_PdS_5_ nanowires. Note that our observation of multi-steps and hysteresis in *I-V* curves in Ta_2_PdS_5_ nanowires is consistent with the scenario generally expected in quasi-1D nanowires as well^17^. At first glance, it seems contrary to the fact that the GL coherence length of Ta_2_PdS_5_, as calculated above and described in Supplementary Note 3, is much smaller than the sample size. However, it has been suggested that for type-II superconductors the required actual size for the quasi-1D superconductivity can be determined by their penetration depth^17,38^, unlike in type-I superconductors where the diameter of the wires needs to be less than its GL coherence length.

We then extract the magnetic properties of Ta_2_PdS_5_ to estimate the penetration depth. The obtained magnetic moment versus magnetic field of Ta_2_PdS_5_ is shown in Fig. 3e, exhibiting a typical behavior of type-II superconductors^39,40^. From the initial magnetization curve (Fig. 3f), *B*_C1_(1.8 K) = 1.8 Oe can be acquired. Using the equation^20^ *B*_C_(*T*) = *B*_C_(0)[1−(*T*/*T*_C_)^2^], we can obtain the low temperature *B*_C1_(0) = 2.6 Oe. Utilizing the *B*_C2a´_(0) = 4.7 T in Supplementary Note 3^39^, the GL parameter can be estimated to be 220 following the equation^20,25^ *B*_*C*2_(0)/*B*_*C*1_(0) = 2*κ*(0)^2^/ln *κ*(0). Using^20,25^ *κ*(0) = *λ*(0)/*ξ*(0), we can obtain the penetration depth *λ*(0) of Ta_2_PdS_5_ nanowire ~1848 nm, which is much larger than the device thickness of 120 nm. Note that the penetration depth we estimated here is similar to other quasi-1D superconductors like Nb_2_PdS_5_ ~785 nm [ref.^41^], Nb_2_PdS_5_~410 nm [ref.^20^] and Sc_3_CoC_4_ ~970 nm [ref.^39^]. Thus, we believe that the superconductivity in Ta_2_PdS_5_ is quasi-1D.

### Superconductor-metal transition and quantum Griffiths state

**Fig. 4:**
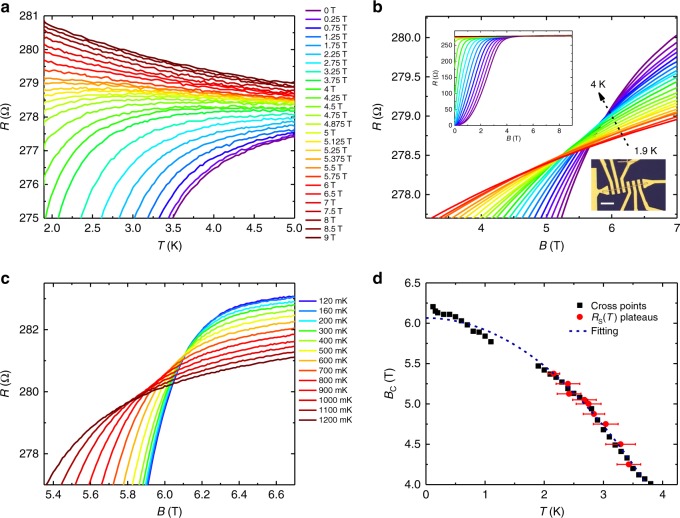
Superconducting-metal transition in Ta_2_PdS_5_ nanowire device with the thickness of 100 nm. **a** Zoom-in view of the temperature-dependent resistance at perpendicular magnetic field ranging from 0 to 9 T, showing an insulating behavior at high magnetic fields. **b** Zoomed magnetoresistance of the device measured at a temperature of 1.9–4 K under perpendicular magnetic field configuration, showing a series of cross points as temperature changes. Inset: up left, the whole range of the magnetoresistance isotherms; down right, an optical image of the device, scale bar 10 μm. **c** Perpendicular magnetoresistance isotherms of the device at various temperatures ranging from 0.12 to 1.2 K. **d** Critical magnetic fields *B*_C_(*T*) extracted from **a**, **b**, and **c**. Black squares are the crossing points of *R*_S_(*B*) curves at every two adjacent temperatures around the transition regime. The red dots are taken from the *R*_S_(*T*) curves at given magnetic fields in **a** where d*R*_S_/d*T* changes sign. Error bars of the red dots represent the temperature uncertainty due to the experimental resolution in **a** where d*R*_S_/d*T* changes sign. The blue dashed line is the theoretical fitting to *B*_C2_(*T*) *=* *B*_C2_(0)[1*−*(*T/T*_C_)^2^]

Next, we study the magnetoresistance of the Ta_2_PdS_5_ in a perpendicular magnetic field configuration. Figure 4a reveals the temperature-dependent resistance of a nanowire device (cross-sectional area 300 nm × 100 nm) under various magnetic fields. With the increase of the magnetic field, the superconducting transition shifts monotonically to lower temperatures similar to Fig. 2c. As the magnetic field continues to increase, the superconductor gradually changes to a localized metal, indicating a magnetic field induced SMT. This SMT behavior is further explored in magnetoresistance isotherms in Fig. 4b where *R*_S_(*B*) curves cross each other. Intriguingly, the crossing point of SMT seemingly changes as the temperature varies. To investigate the SMT behavior in Ta_2_PdS_5_ nanowires, we measure the sample in the dilution temperature environment. Figure 4c shows the magnetoresistance isotherms of the device under an ultra-low temperature ranging from 0.12 to 1.2 K. A series of crossing points have also been observed. We summarize the crossing points in both high and low-temperature regimes in Fig. 4d, where the black squares are crossing points of every two adjacent *R*_S_(*B*) curves. Also, as shown by the red dots in Fig. 4d, the *R*_S_ plateaus where d*R*_S_(*T*)/d*T* changes sign for a given magnetic field have been extracted from *R*_S_(*T*) curves in Fig. 4a. We have also used the empirical equation to fit the crossing points (blue dashed line in Fig. 4d). In the high-temperature regime, the data fit the equation well; while in the ultra-low-temperature region, *B*_C_ diverges from the tendency of the empirical equation (*T* *<* 0.7 K). The experimental data of *B*_C_(0) > 6.2 T is substantially larger than the fitted result from the empirical equation (*B*_C_(0) = 6.06 T).

Typically, for SIT, the critical resistance corresponding to the border between the superconducting and insulating region at low temperatures should be the quantum unit of resistance (h/4e^2^ ∼ 6450 Ω), and the critical exponent remains to be a constant^42^. Theoretical investigations^43^ show that unpaired electrons could originate from the pair-breaking mechanism of dissipation effect, giving rise to a much smaller critical resistance than h/4e^2^. This explains our experimental data that the critical resistance of Ta_2_PdS_5_ nanowire is much smaller than the quantum resistance h/4e^2^. More recently, theoretical studies also show that quenched disorders could dramatically change the scaling behavior of SMT and result in an activated scaling behavior identical to that of the quantum random transverse-field Ising model^44^. In that system, the activated scaling behavior, called quantum Griffiths singularity, exhibits continuously varying dynamical exponent *z* when approaching the infinite-randomness quantum critical point. Recent experiments on 2D superconducting Ga thin film, monolayer NbSe_2_ and ionic liquid gated ZrNCl and MoS_2_ have envisaged the similar continuous line of “critical” points and the divergence of the dynamical critical exponent in SMT, which experimentally reveals the existence of quantum Griffiths singularity state in SMT in 2D superconductors^12,14^.

**Fig. 5:**
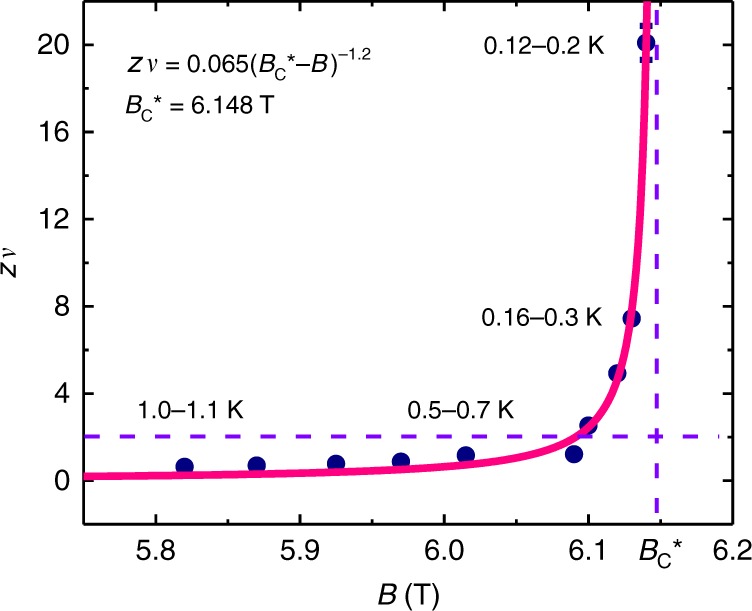
The activated quantum scaling behavior in Ta_2_PdS_5_. Deduced exponent *zν* as a function of magnetic field, showing no sign of saturation when approaching the zero-temperature limit. The solid pink line is the fit based on the activated scaling law equation as shown in the figure, two violet dashed lines represent the constant values with *B*_C_* = 6.148 T and *zν* = 2. The error bar representing the width of *zv* value was acquired during the scaling analysis performed in Supplementary Figure 11

Inspired by the new discovery of quantum Griffiths singularity in SMT, we analyze multiple crossing points in Ta_2_PdS_5_ nanowires using the scaling analysis method, according to which the resistance in the vicinity of a critical point follows^7,45^

2 $${R}\left( {\delta ,T} \right) = R_{\mathrm{C}}f\left( \delta T^{ - 1/zv} \right),$$

where the *δ *= |*B* − *B*_C_| is the deviation from the critical field, *R*_C_ is the critical resistance, *f*(*x*) is the scaling function with *f*(0) = 1, *δ* is tuning parameter, *z* is the dynamic critical exponent, and *ν* is the correlation length critical exponent. One “critical” point (*B*_C_, *R*_C_) of a certain small critical transition region is defined as the crossing points of several adjacent *R*_S_(*B*) curves (See Supplementary Figure 11 for details). The deduced magnetic field dependence of the effective “critical” exponent *zν* is shown in Fig. 5. In the relative high-temperature regime, *zν* value increases slowly with the magnetic field, while in the ultra-low-temperature regime, *zν* grows quickly as the temperature decreases. Here we note that one *zν* value corresponds to a temperature region rather than a certain temperature. Next, we try to fit the extracted *zν* values versus B using the activated scaling law *zν* = *C*|*B*_C_* − *B*|^−*ηψ*^, where *C* is a constant, *η* ≈ 1.2 is the correlation length exponent and *ψ* ≈ 1 is the tunneling critical exponent in quasi-one dimension^12,14,46^. The activated scaling law fits the data well (the solid pink line in Fig. 5, where *B*_C_* = 6.148 T), indicating the existence of infinite-randomness quantum critical points in Ta_2_PdS_5_ nanowires. As mentioned before, the enhanced quenched disorders^13^ at low temperatures are the main inducement of the quantum Griffiths singularity in Ta_2_PdS_5_. Although the Ta_2_PdS_5_ nanowires are single crystalline with good uniformity, there may still be some defects. In addition, the interface effect coming from the device fabrication and scattering from the substrate could also introduce disorders in the system. All of these above could be the origin of the enhanced quenched disorders at low temperatures^14^, resulting in the infinite *zν* value when the critical point *B*_C_* is approached.

## Discussion

With the reduced dimensionality of angle-dependent *B*_C2_ of thin Ta_2_PdS_5_ nanowires, the appearance of multiple *I-V* steps, the enhanced Ohmic finite resistance when the thickness of the device reduces, the good fittings of the temperature-dependent critical current to the Bardeen’s formula^37,38^ for quasi-1D superconductors, and the *zν* value to the 1D equation^46^ *zν* = *C*|*B*_C_* − *B*|^−1.2^, the quasi-1D superconductivity is suggested to present in Ta_2_PdS_5_ nanowires. Considering the crystal lattice constants^19^ *a* = 1.2 nm, *b* = 0.3 nm, and *c* = 1.5 nm, the GL coherence lengths along different crystal axes are larger than the lattice constants (*ξ*_a´_(0) = 8.4 nm, *ξ*_b_(0) = 4.2 nm, and *ξ*_c´_(0) = 8.6 nm, see Supplementary Note 3 for details). Note that the GL coherence length along different directions is also smaller than our nanowire thickness (~120 nm). However, the actual effective superconducting thickness of Ta_2_PdS_5_ nanowire should be smaller than the sample thickness^3^, which may make the superconductivity in Ta_2_PdS_5_ nanowires quasi-1D. Nevertheless, the penetration depth of ~1848 nm calculated above is significantly larger than the sample thickness, satisfying the condition required for quasi-1D superconductivity in type-II superconductors^17,38^. Also, from previous experiments on quasi-1D superconducting nanowires, the diameter of the systems ranges from 10 to 1000 nm^32,34,38,47–49^ and our sample thickness is within that range. We need to further mention that the unique weak coupled Ta-S chains^19,27,50^ along the *b*-axis, which are responsible for the superconductivity also make the superconductivity in Ta_2_PdS_5_ nanowires quasi-1D^39,51^. Considering all the facts listed above, we conclude that the superconductivity in Ta_2_PdS_5_ nanowire is quasi-1D in nature (also see Supplementary Note 5 for all listed facts for quasi-1D superconductivity in Ta_2_PdS_5_ nanowires).

The observation of quantum Griffiths singularity state in Ta_2_PdS_5_ also suggests that the critical phenomenon below the quantum critical point is described by exponentially small but nonzero probability of large-ordered regions-rare regions, which can be viewed as the superconducting puddles surviving in the normal state background with a long time and length scale as the temperature approaches 0 K^15,52^. Instead of one or two crossing points in the magnetoresistance isotherms cross in the SIT or SMT, recent observations of the quantum Griffiths singularity in thin Ga^12^ films and monolayer NbSe_2_^14^ have shown one cross point in the high-temperature regime and a series of crossing points in the ultra-low-temperature regime. Interestingly, our study in quasi-1D Ta_2_PdS_5_ nanowire has shown that the crossing point moves continuously in both high- and low-temperature regimes. As a result, the dynamical exponent *z* continuously varies in both high- and low-temperature regimes. The reason for this may be due to the fact that the thermal fluctuations at such a temperature regime (2–4 K) are unable to smear out the inhomogeneity caused by quenched disorders^12,53,54^. However, the exquisite physics in this system needs further theoretical and experimental investigations.

In summary, we have demonstrated the systematic study of magnetotransport properties of quasi-1D Ta_2_PdS_5_ nanowires. The strong anisotropic superconducting behavior and hysteretic multiple voltage steps in its *I-V* relation indicate its typical quasi-1D nature in superconductivity. Importantly, the nanowire undergoes a SMT and shows signatures of quantum Griffiths singularity state when approaching zero-temperate quantum critical point. These findings shed lights on the understanding of the superconductor-metal and metal-insulator transitions. In addition, the appealing physical properties unveiled in this study demonstrate Ta_2_PdS_5_ to be a promising platform for possible applications in quantum computing devices.

## Methods

### Sample preparation

Single crystals of Ta_2_PdS_5_ were synthesized by the CVT method using iodine as a transport agent. Before the crystal growth, a quartz tube containing iodine and the stoichiometric ratio of Ta, Pd, and S powders with 1% excess of S was evacuated and sealed. The sealed tube was then placed in a double zone furnace horizontally and grew for 2 weeks with the temperature gradient of 775–850 ^o^C, after which needle-like single crystals of Ta_2_PdS_5_ were formed at the low-temperature end.

### Material characterizations

The structural and compositional characteristics of the nanowires were investigated using TEM (FEI Tecnai F20, 200 kV, equipped with EDS). The nanowires were dry transferred onto Lacey carbon films supported by a copper grid.

### Device fabrication

Ta_2_PdS_5_ nanowires with different thickness were obtained through mechanical exfoliation onto pre-patterned SiO_2_(285 nm)/Si substrates from bulk crystals. The electrical contacts of Ta_2_PdS_5_ devices were fabricated along the *b*-axis of the crystal by EBL using Polymethylmethacrylate/Methyl methacrylate bilayer polymer. Ti/Au (5 nm/150 nm) electrodes were then deposited using magnetron sputtering.

### Transport measurements

Four-terminal temperature-dependent magnetotransport and *I-V* measurement measurements were carried out in a Physical Property Measurement System (PPMS) system (Quantum Design) using lock-in amplifier (SR830), Agilent 2912 and Keithley 2182. Ultra-low-temperature magnetotransport measurements were performed in an Oxford 3He/4He-dilution refrigerator equipped with a superconducting magnet using SR830.

## Electronic supplementary material

Supplementary Information

Description of Additional Supplementary Files

Supplementary Data 1

## Data Availability

All of the experimental data supporting this study are available from the corresponding author.
