# Supplementary material for: Signature of quantum Griffiths singularity state in a layered quasi-one-dimensional superconductor
Source: Nat Commun. 2018 Nov 7;9:4656. doi: 10.1038/s41467-018-07123-y (PMC6220168; doi:10.1038/s41467-018-07123-y)
Supplement: Supplementary file 1 — Supplementary Information [file 41467_2018_7123_MOESM1_ESM.pdf]

Supplementary Information for

Signature of quantum Griffiths singularity state in a layered quasi-one-dimensional superconductor

Zhang et al.

Supplementary Figures

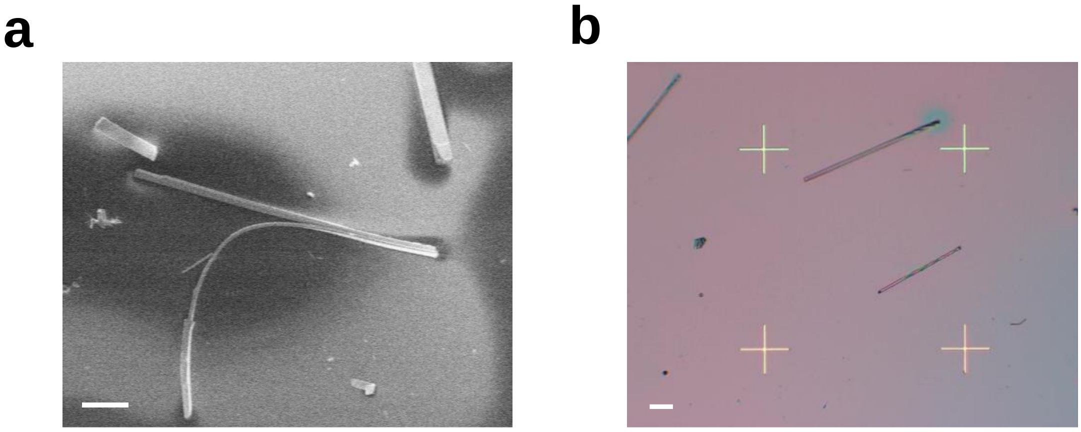

Supplementary Figure 1| Images of Ta_2PdS_5 nanowires after exfoliation. (a) SEM image of Ta_2PdS_5 nanowires exfoliated on PDMS substrate showing cleavage edges, scale bar, 4 um. **(b)** Optical image of Ta_2PdS_5 nanowires exfoliated on pre-patterned SiO_2 (285nm)/Si substrate, scale bar, 15 μm.

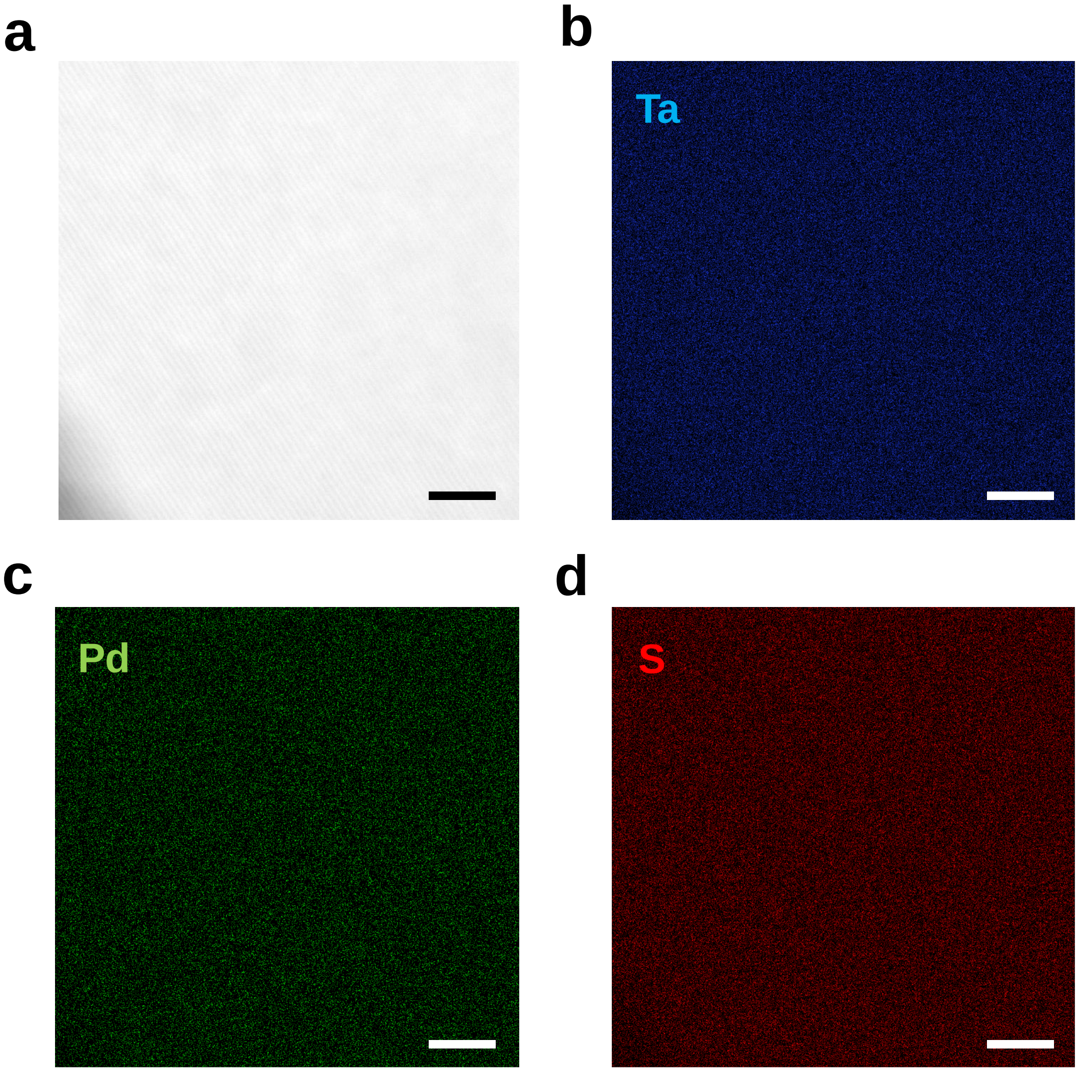

Supplementary Figure 2| EDS mapping for the local crystal region with comparatively uniform projected thickness. (a) HAADF image taken from the tip region of a Ta_2PdS_5 crystal. Scale bar, 5nm. **(b)- (d)** Corresponding EDS elemental maps for (b) Ta, (c) Pd and (d) S. Scale bars, 5nm.

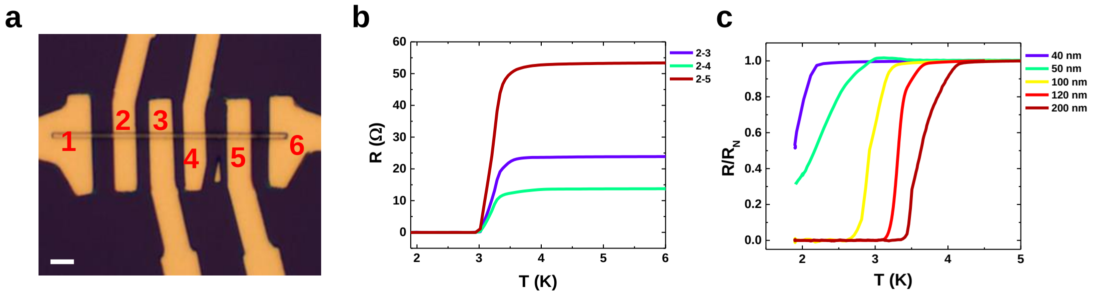

Supplementary Figure 3| Length-dependent superconducting properties in the same nanowire device and T_C of devices with various thickness. (a) Optical image of Ta_2PdS_5 multiple contact nanowire device. Scale bar, 4 μm. **(b)** *R*-*T* curves around the transition temperature of different length of the device in (a), showing the same T_C of 3.2 K. These results indicate that the nanowire is in good uniformity and the proximity effect from the contact is negligible[1]. **(c)** Thickness-dependent T_C of Ta_2PdS_5 nanowires with a width around 300 nm.

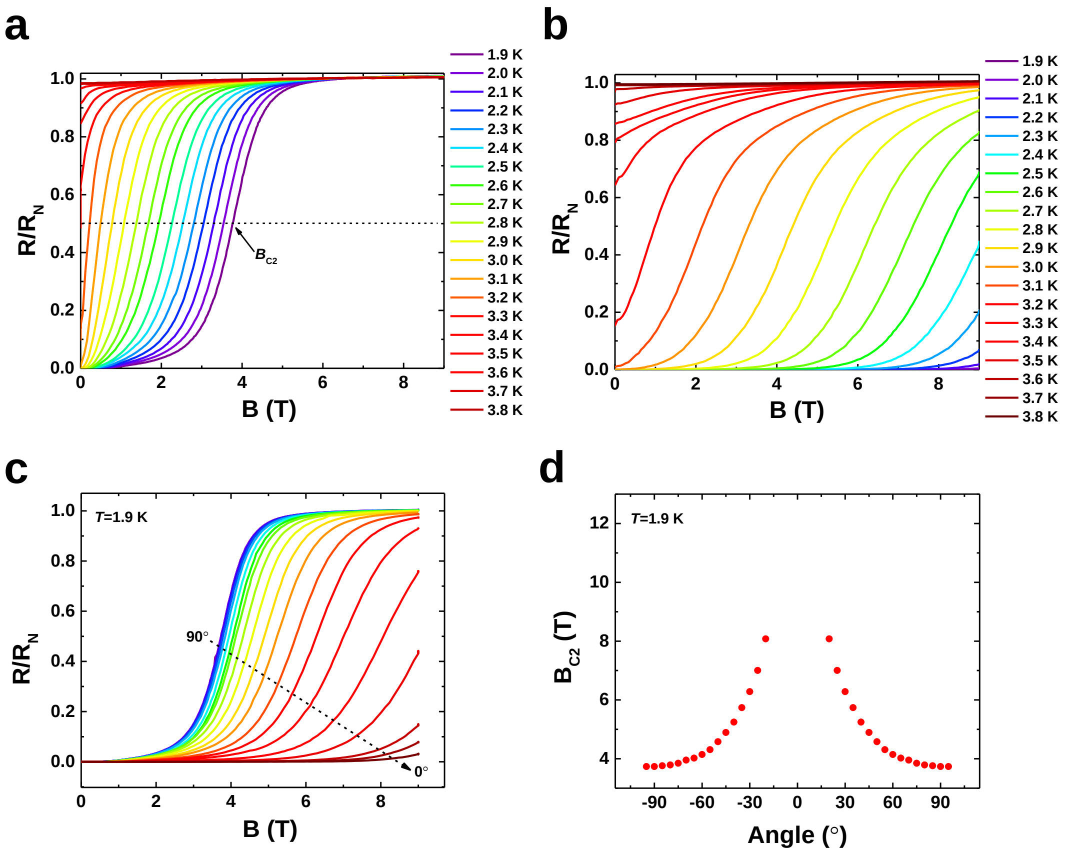

Supplementary Figure 4| Additional magnetoresistance data of device 01. (a) Perpendicular magnetoresistance of the device in Figure 2 in the main text at various temperatures. **(b)** Magnetoresistance isotherms under parallel magnetic field configuration. **(c)** Angle-dependent magnetoresistance at T=1.9 K. **(d)** Deduced B_{C2} as a function of θ at T=1.9 K, showing a very large anisotropy.

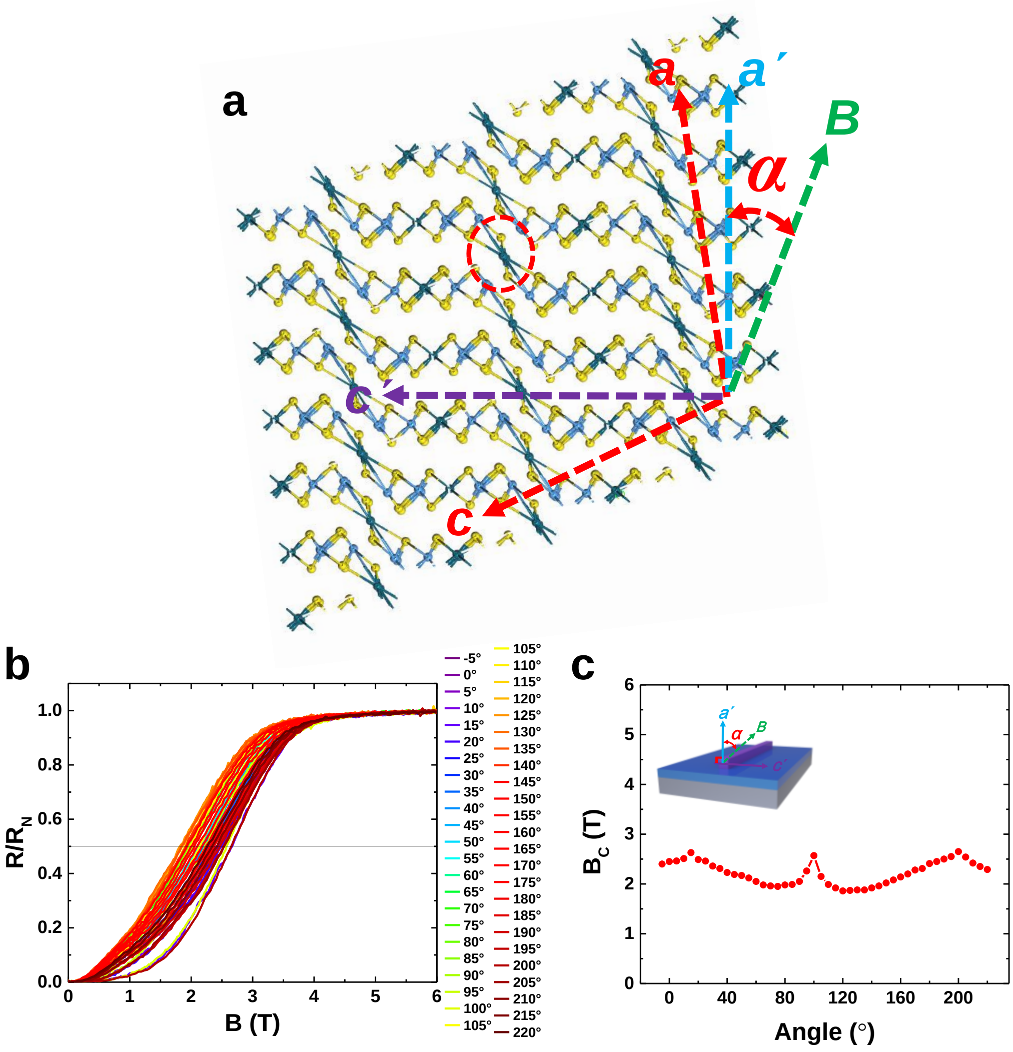

Supplementary Figure 5| Anisotropy of B_{C2} in the *a*-*c* plane. (a) Crystal structure of Ta_2PdS_5 in *a*-*c* plane, **a** and **c** are the actual crystal axis of Ta_2PdS_5. **c´** (red dashed arrow) stands for the direction of the cleavage edges. α stands for the angle between the applied magnetic field and **a´** direction (blue dashed arrow). **(b)** Angle-dependent B_{C2} of a Ta_2PdS_5 nanowire device at T=1.9 K. The device thickness is 120nm. (c) Deduced B_{C2} as a function of angle α. Inset is a schematic configuration of angle-dependent magnetoresistance measurements in *a*-*c* plane. The α=0 here is defined as the magnetic field parallel to the a´-axis in **(a)**.

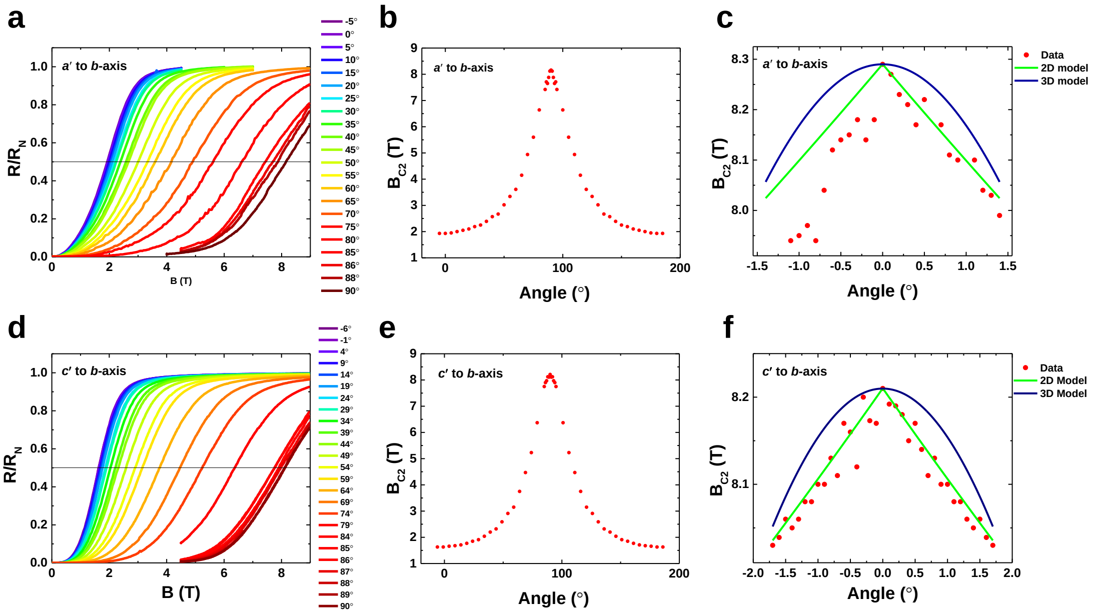

Supplementary Figure 6| (a)(d) Angle-dependent magnetoresistance of Ta_2PdS_5 nanowire device at T=2.4 K when the magnetic field changes from *a*′-/*c*′- to *b*-axis, respectively. The device thickness is 120nm. **(b)(e)** Deduced B_{C2} as a function of angle α, exhibiting similar behavior in these two geometries. **(c)(f)** B_{C2} as a function of angle θ when the magnetic field is nearly parallel to *b*-axis. The blue and green lines are the fitting based on 3D and 2D model, where none of them agrees with the experimental data.

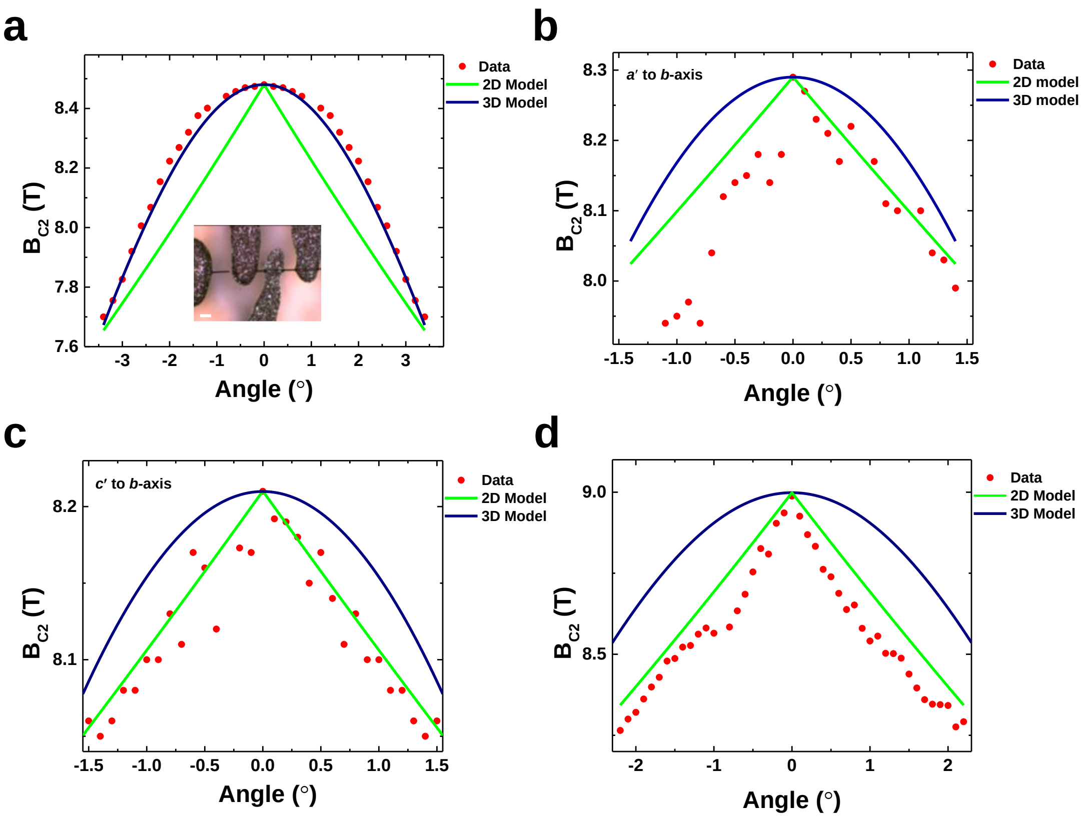

Supplementary Figure 7| Anisotropic B_{C2} of Ta_2PdS_5 with different thickness. (a) Deduced B_{C2} as a function of angle θ with magnetic field nearly along *b*-axis, showing a good fitting to 3D model (blue line). The green line is fitting to the 2D model and the device thickness is ~5μm (Bulk). Inset in (a), an optical image of the Ta_2PdS_5 bulk device, the contacts are made of silver paint. Scale bar, 50μm. **(b)(c)** Deduced B_{C2} as a function of angle θ with magnetic field nearly along *b*-axis, showing no good fittings to either 3D or 2D model. The device thickness is 120nm. **(d)** B_{C2} as a function of angle θ when the magnetic field is nearly parallel to *b*-axis, showing no good fitting to either 3D or 2D model. The device thickness is 110nm.

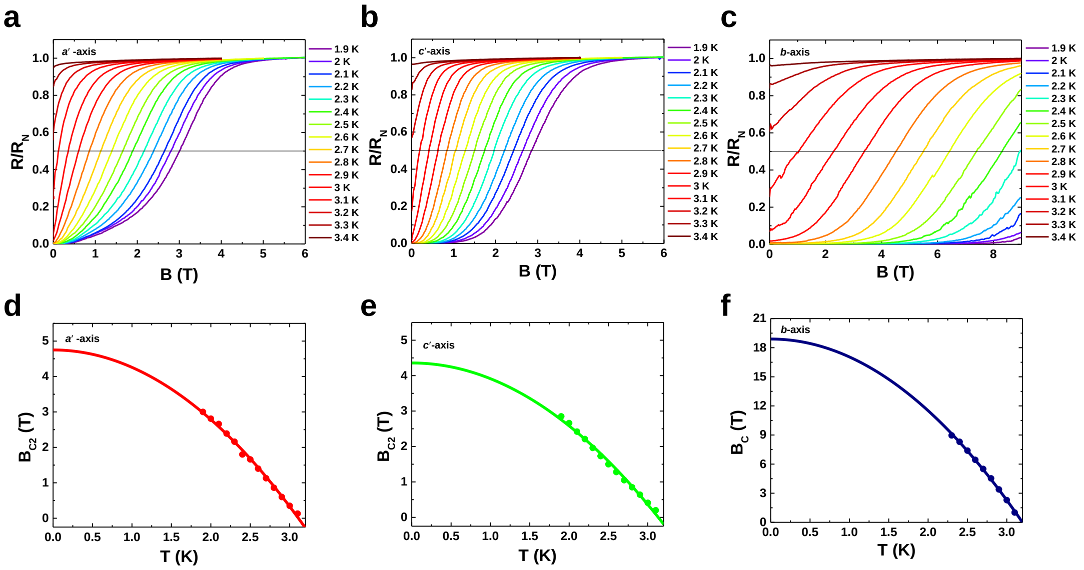

Supplementary Figure 8| Magnetoresistance isotherms of the Ta_2PdS_5 nanowire with the magnetic field along different crystal axis. (a)-(c) Magnetoresistance isotherms of Ta_2PdS_5 nanowire when the magnetic field is along **a′**, **b** and **c′**, respectively. **(d)-(f)** Deduced B_{C2} as a function of temperature when the magnetic field is along **a′**, **b** and **c′**, respectively. The solid lines are fitting to the equation $B_{C2}(T)=B_{C2}(0)[1-(T/T_C)^2]$.

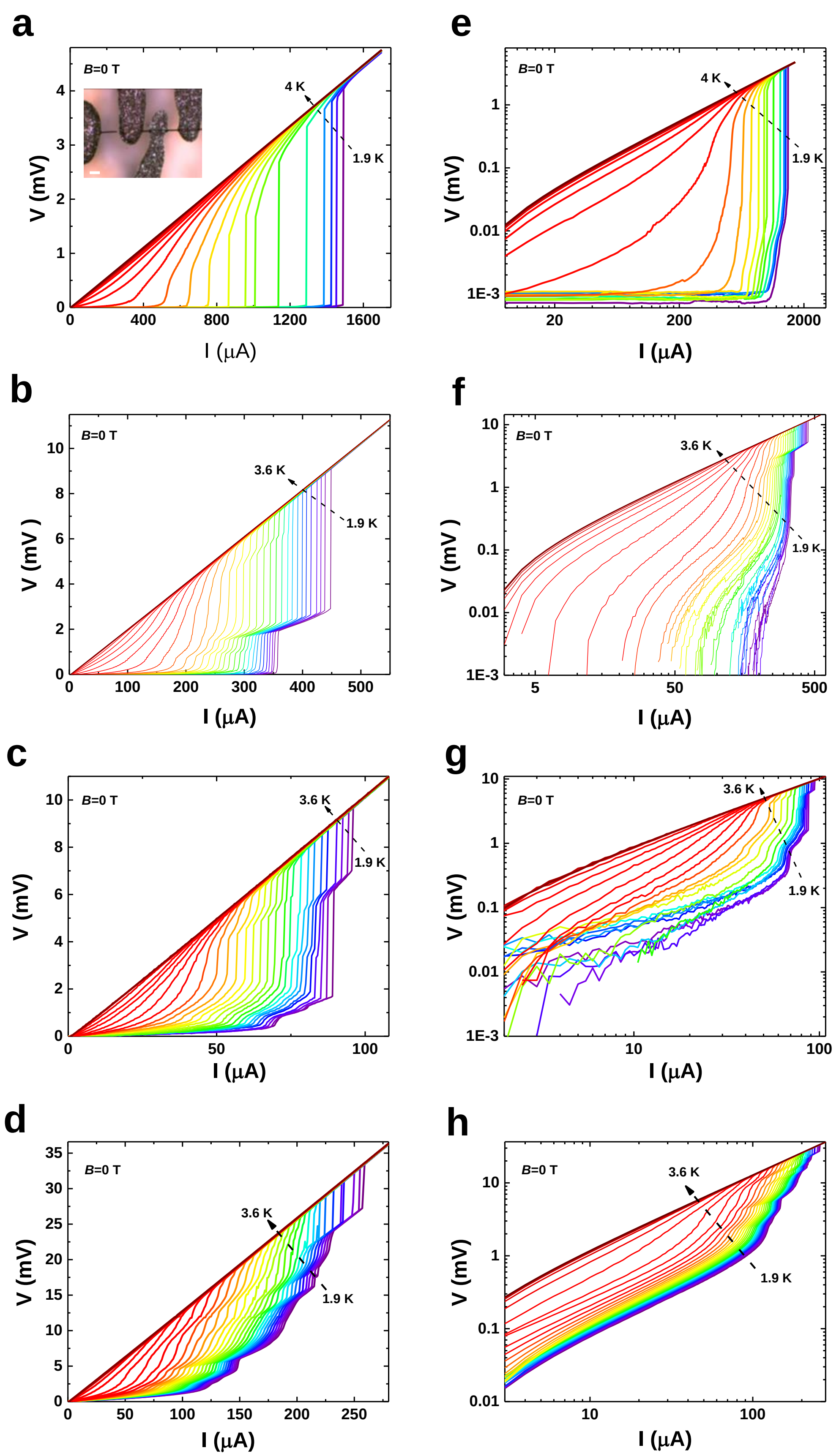

Supplementary Figure 9| *I-V* characteristics of Ta_2PdS_5 with various thickness.

(a)-(d) Linear scale *I-V* characteristics of the Ta_2PdS_5 nanowire with the thickness of ~5μm (Bulk), 220nm, 120nm and 110nm, respectively. Inset in (a), an optical image of the Ta_2PdS_5 bulk device, the contacts are made of silver paint. Scale bar, 50μm. **(e)-(h)** Logarithmic scale *I-V* characteristics of the Ta_2PdS_5 nanowires with the thickness of ~5μm (Bulk), 220nm, 120nm and 110nm, respectively.

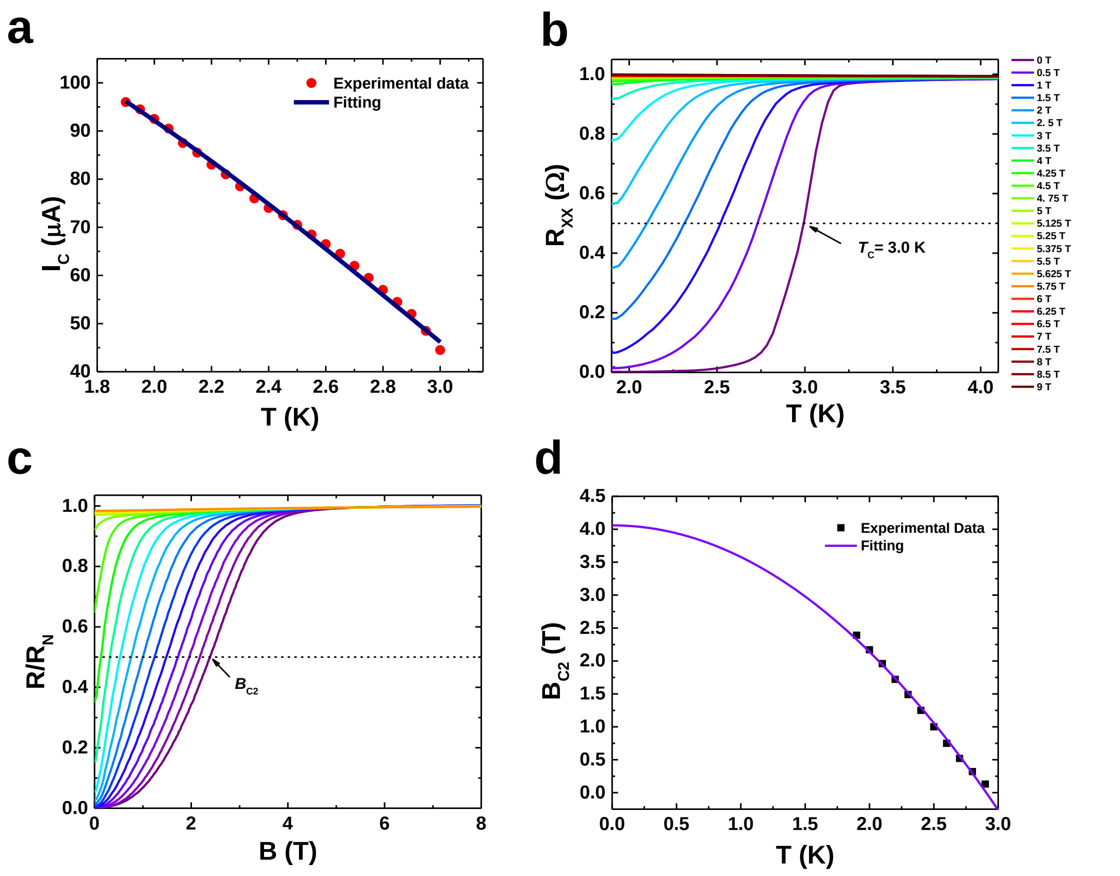

Supplementary Figure 10| Temperature-dependent critical current and additional data of device 02. (a) Temperature-dependent critical current deduced from main text Figure 3a. The blue solid line is the fit of the temperature-dependent critical current to the Bardeen's theoretical formula[2, 3] for quasi-1D superconductors $I(T) = I_c(0)(1 - (T/T_{C0})^2)^{3/2}$, where T_{C0} is the transition temperature T in the absence of currents and fields. **(b)** *R*-*T* curves of the device under several perpendicular magnetic fields of the device 02 in Figure 4 of the main text, the dashed line depicts the definition of T_C. **(c)** Definition of B_{C2} (dashed line), which corresponds to the middle point of the normal resistance[4]. **(d)** Deduced temperature dependent B_{C2}, the solid purple line shows the fit to the equation $B_{C2}(T)=B_{C2}(0)[1-(T/T_C)^2]$. We have used the same equation to fit the B_{C2} and the crossing points of magnetoresistance isotherms in Figure 4 (main text)[5, 6 7].

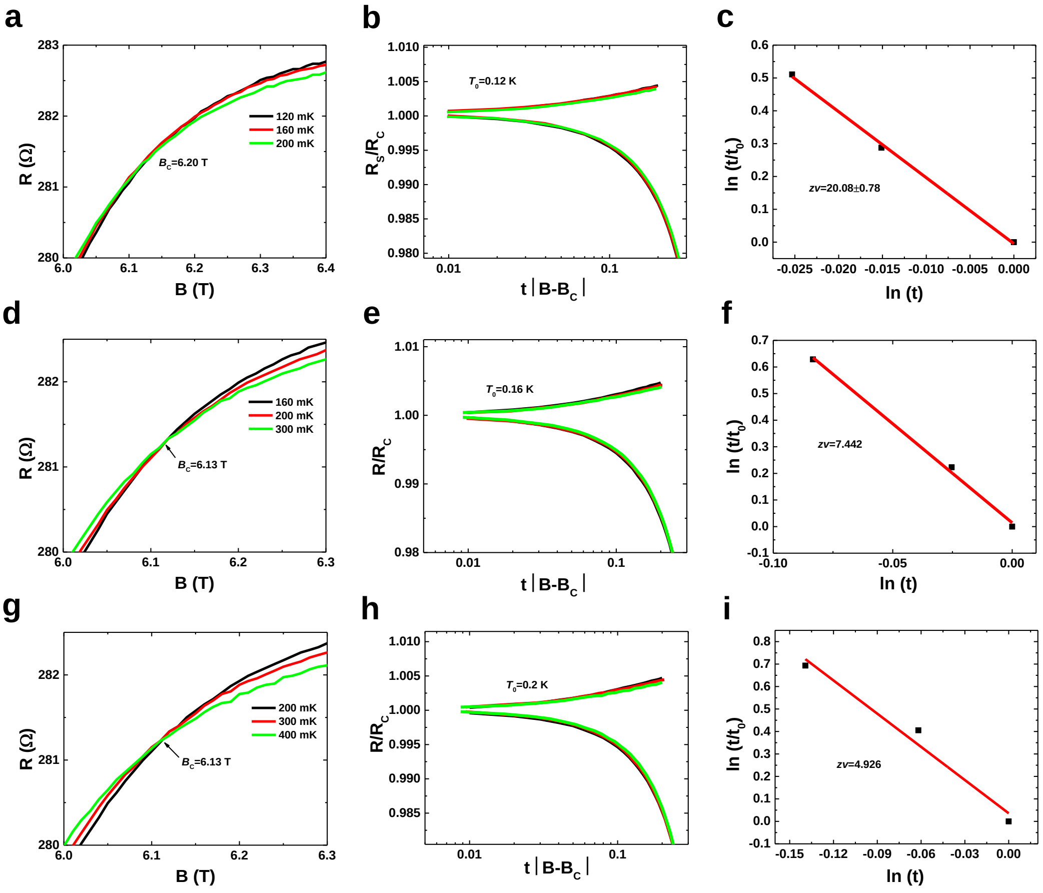

Supplementary Figure 11| Detailed analysis of the extraction of the critical exponent. (a)(d)(g) The magnetoresistance close to the superconductor to metal boundary at different temperatures. Three adjacent curves were chosen. **(b)(e)(h)** Normalized resistance R_S versus scaling variable[5, 6] $t|B-B_X|$, where $t = (T/T_0)^{-1/zv}$, B_X and T_0 are the critical magnetic field and the lowest temperature in (a)(d)(g), respectively. **(e)(f)(i)** ln(t) as a function ln(t/t0), where the critical exponent zv can be calculated through the linear fitting (red solid line).

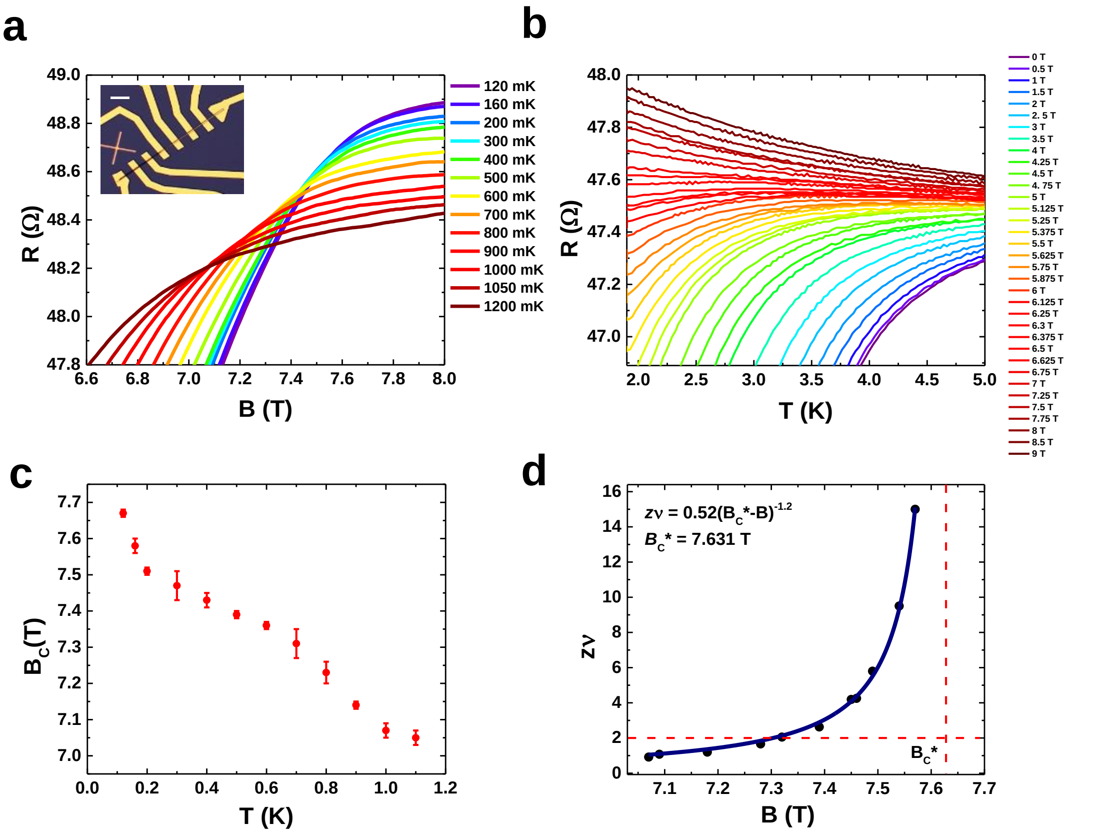

Supplementary Figure 12| Magneto-transport measurements and analysis on another Ta_2PdS_5 device. (a) Magnetoresistance isotherms of the device at various temperatures from 0.12 to 1.2 K under perpendicular magnetic field configuration, where an apparent crossing region can be observed. Inset, an optical image of the device, scale bar, 4 μm. The sample thickness is 150 nm. (b**)** Temperature-dependent resistance at the perpendicular magnetic field from 0 to 9 T, showing an insulating behavior at high magnetic fields. **(c)** Critical magnetic fields B_C(T) extracted from (a). Error bars represent the B_C(T) uncertainty between two adjacent magnetoresistance isotherms.**(d)** Calculated exponent $z\nu$ (blue dots) versus magnetic field and the corresponding temperature region in (a), exhibiting no sign of saturation (consistent with the device

shown in the main text) when approaching the zero-temperature limit. The solid blue line is the fit based on the activated scaling law equation shown in the figure.

Supplementary Note 1. The reason for the broadened R-T and notes on Ta_2PdS_5 crystal quality.

As shown in Supplementary Figure 3c, as the thickness of the nanowire goes to thinner, its T_C drops and the broadening of the superconducting transition emerges. Note that when the superconductor reduces to a lower dimension, the broadening of the superconducting transition emerges because of the thermal activated or quantum activated phase slip[8] and enhanced thermal fluctuations[4]. Previously, the broadening of the superconducting transition has been observed both in quasi-1D Sn nanowires[8] and 2D atomically thin $NbSe_2$[9]. The difference of the two superconducting systems is that for the quasi-1D system it has *I-V* steps[8] while for 2D atomically thin $NbSe_2$ it exhibits broadened superconducting transition and its *I-V* characteristics show BKT-like behavior[9]. Thus, the distribution of transition temperatures in Ta_2PdS_5 is the indication of quasi-1D of its superconductivity rather than the inhomogeneous crystal quality of the sample.

It is noteworthy that the pseudo-color variation exhibited within the EDS mapping region (main text Figure 1f-h) is mainly due to the thickness difference (main text Figure 1c). To carefully examine the crystal quality, we further conducted the EDS mapping for the local crystal region with comparatively uniform projected thickness, using scanning transmission electron microscopy (STEM). Supplementary Figure 2 shows the high-resolution high angle annular dark-field (HAADF) image and the corresponding EDS maps. As can be seen, the Ta_2PdS_5 crystal we used is chemically homogeneous and is single crystalline with high quality.

Supplementary Note 2. The anisotropy of B_{C2} in Ta_2PdS_5 with a thickness of ~5μm (bulk) to 110nm.

We have measured the anisotropy of B_{C2} as the magnetic field changes from perpendicular to parallel to *b*-axis, the same configuration as we measured in the main text. Supplementary Figures 6a and d are the angle-dependent magnetoresistances of the device when the magnetic field changes from a'/c' to *b*-axis direction, respectively. The deduced B_{C2} relations with angle θ (same as the θ in the main text) are shown in Figures 6b and d, where they exhibit very large anisotropy. We have also measured B_{C2} with the magnetic field nearly along b-axis under these two circumstances, and the data are shown in Supplementary Figures R6c and f, respectively. The angle-dependent B_{C2} at small α shows very different characteristics from 2D superconductors such as ionic gated MoS_2 and SnS_2[10, 11]. We have tried to fit the angle-dependent B_{C2} data to both 2D Tinkham formula[11, 12],

$$\left(\frac{B_{C2}(\theta)cos(\theta)}{B_{C2//}}\right)^2 + \left|\frac{B_{C2}(\theta)sin(\theta)}{B_{C2\perp}}\right| = 1,$$

and 3D anisotropic Ginzburg-Landau model[10, 13],

$$B_{C2}(\theta) = \frac{1}{\sqrt{(acos\theta)^2 + (bsin\theta)^2}},$$

where $B_{C2//}$ and $B_{C2\perp}$ are upper critical filed at θ=0◦ and θ=90◦, respectively. We find that neither 3D (blue line) nor 2D (green line) model fits the experimental data well.

We further carried out angle-dependent magnetoresistance measurements on samples with different thickness. Supplementary Figure 7 presents are the angle-dependent B_{C2} around θ=0. Here, θ is the angle between the magnetic field and *b*-axis of the crystal, the same as the definition of θ in the main text. We fit the data using both 3D and 2D models. The angle-dependent B_{C2} ~5μm (Bulk) sample shows a very good fit to the 3D model, which is consistent with the previous study[14]. However, the angle-dependent B_{C2} of thin samples do not fit well with either 3D or 2D model. As far as we know, up to now, there is no theoretical models/equations that can be used for the data fitting in the quasi-1D superconducting system. And at this point, the data we acquired clearly

show a large B_{C2} anisotropy with respect to *b*-axis in Ta_2PdS_5.

In the above section, we have measured the angle-dependent B_{C2} of Ta_2PdS_5 with a thickness from ~5μm (bulk) to 110nm. We find that for bulk samples its angle-dependent B_{C2} fits the 3D model very well while for thin samples B_{C2} fits neither 3D nor 2D model, This provides another evidence of the quasi-1D superconductivity in Ta_2PdS_5 nanowires.

Supplementary Note 3. The calculation of zero-temperature superconducting coherence lengths along different crystallographic axes.

It is important to know the relation between the actual size of the sample and GL phase coherence length along all three crystallographic axes. Thus, we carried out experiments to measure the zero-temperature GL coherence length along three crystallographic axes. To achieve this goal, we first measure the anisotropy of B_{C2} in the *a*-*c* plane of the Ta_2PdS_5 nanowire. Supplementary Figure 5a shows the crystal structure of Ta_2PdS_5 in the *a*-*c* plane. The relative weak Pd-S bonds (red dashed circles) make **c′** direction (purple dashed arrow) the cleavage edges[14]. We define the angle between the applied magnetic field direction and *a′* (blue dashed arrow) as α. As shown in Figure 5b, we rotate the sample in the *a*-*c* plane of Ta_2PdS_5 (α varies) and obtain the angle-dependent magnetoresistance of the Ta_2PdS_5 nanowire. The deduced B_{C2} of the device is shown in Supplementary Figure 5c, where it exhibits smaller anisotropy compared to that with respect to *b*-axis. This is in fact not surprising because for quasi-1D nanowire superconductor the anisotropy along the nanowire direction (b-axis of Ta_2PdS_5) is typically more evident[15, 16], similar to the scenario of 2D superconductors where in-plane anisotropy is almost negligible compared to out-of-plane one[6, 11].

Then, we calculate the zero-temperature superconducting coherence lengths along different crystal axes. Supplementary Figures R8 (a)-(c) are the magnetoresistance isotherms of the device when the magnetic field is along **a′**, **c′** and **b**, respectively. The deduced B_{C2} with respect to temperature is shown in Supplementary Figure 8 (d)-(f). After fitting the data using equation $B_{C2}(T)=B_{C2}(0)[1-(T/T_C)^2]$, we extracted the $B_{C2}(0)$ for **a′**, **b** and **c′** to be 4.7, 19.0 and 4.4 T, respectively. Using the equation $B_{c2}(0) = \Phi_0/[2\pi\xi(0)]^2$, we can calculate the Ginsburg-Landau (GL) coherence length along different directions, that is, $\xi_{a'}(0)$=8.4nm, $\xi_b(0)$= 4.2nm, and $\xi_{c'}(0)$=8.6nm. Considering the small B_{C2} anisotropy in the a-c plane, the GL coherence length in a and c-axis should be all around 8.4 nm. Considering the crystal lattice constants[17] *a*=1.2nm, *b*=0.3nm, and *c*=1.5nm, the GL coherence lengths along different crystal axes are larger than the lattice constants. Note that the GL coherence length along different directions is also

smaller than our nanowire thickness. However, the actual effective superconducting thickness of Ta_2PdS_5 nanowire should be smaller than the sample thickness[18], which may make the superconductivity in Ta_2PdS_5 nanowires quasi-1D. Also, from previous experiments on quasi-1D superconducting nanowires, the diameter of the systems ranges from 10-1000nm[3, 8, 19-22], and our sample thickness is within that range. We need to further mention that the unique weak coupled Ta-S chains[14, 17, 23] along the *b*-axis which are responsible for the superconductivity can also make the superconductivity in Ta_2PdS_5 nanowires quasi-1D[24, 25].

Supplementary Note 4. The reasons for the appearance of multiple I-V steps in Ta_2PdS_5 nanowires.

As for the multiple *I-V* steps in the *I-V* characteristic in Ta_2PdS_5. It is known that *I-V* steps occur in quasi-1D superconducting channels like nanowires[8, 26] and whiskers[27] which exhibit phase slip centers[3]. We have performed the *I-V* measurement of Ta_2PdS_5 nanowires with different thickness. Supplementary Figure 9 presents the measured *I-V* characteristics in the liner (a)-(d) and logarithmic (e)-(h) scale of Ta_2PdS_5 nanowires with thickness of ~5μm (Bulk) (a)(e), 220nm (b)(f), 120nm (c)(g) and 110nm (d)(h). From the *I-V* curves, we can see that there is only one step in ~5μm (Bulk) thick samples, while as the thickness goes thinner, more and more *I-V* steps emerge. Also, we find that in ~5μm (Bulk) sample at a low temperature of 1.9 K the voltage is zero under low current bias until the current reaches a certain value I_C (note that the data points in Supplementary Figure 9(a) at V~1μV are the noise approaching the measurement limit). For the 220nm sample, there has been some residual resistance below the voltage jump which can be seen in the logarithmic scale in Supplementary Figure 9f. For 120 and 110nm thick sample, also, there has been some residual resistance at low current bias, especially in 110nm sample, the *I-V* relation at low current bias is almost Ohmic (Supplementary Figure 9(d)). Note that this phenomenon is quite the similar to superconducting Sn nanowires[8]. The enhanced Ohmic finite resistance found in the low excitation current limit with a reduced thickness is mainly due to the 1D confinement effect as predicted by a thermal-active phase-slip model which suggests an activated phase-slip process over an energy barrier that brings residual resistance[28, 29]. The fact that multiple *I-V* steps and low current residual resistance only happen in relatively thin samples also suggests that the multiple *I-V* steps in thin Ta_2PdS_5 nanowires should come from the quasi-1D superconductivity rather than the inhomogeneity in the scale of the submicron size of the sample. As shown in Supplementary Figure 10a, we have also fit the temperature-dependent critical current of the Ta_2PdS_5 nanowires (data extracted from main text Figure 3a) to the Bardeen's theoretical formula[2, 3] for quasi-1D superconductors $I(T) = I_c(0)(1-(T/T_{C0})^2)^{3/2}$, where T_{C0} is the transition

temperature T in the absence of currents and fields. We found that the experimental data fits the equation quite well, which serves as another evidence for the quasi-1D superconductivity in Ta_2PdS_5 nanowires.

In short, we have carried out *I-V* measurements on Ta_2PdS_5 with a thickness from 5μm (bulk) to 110nm. The *I-V* characteristics of the bulk Ta_2PdS_5 only show one step, while the *I-V* of the Ta_2PdS_5 nanowire exhibit multiple steps during the phase transition from superconducting to the normal state, which is attributed to the phase-slip process in quasi-1D superconductors[8, 30]. As the thickness of the Ta_2PdS_5 nanowire goes thinner, the enhanced Ohmic finite resistance emerges because of the 1D confinement effect consistent with the thermal-active phase-slip model[8, 28, 29]. Also, the temperature dependent-temperature dependent critical current of the Ta_2PdS_5 nanowires fits the Bardeen's theoretical formula[2, 3] for quasi-1D superconductors very well. All of the above facts suggest the quasi-1D superconductivity in Ta_2PdS_5 nanowires.

Supplementary Note 5. Several evidence supporting the quasi-1D superconductivity in Ta_2PdS_5 nanowires.

Base on the experiments and analysis in the main text and supplementary information above, we've provided several evidence to support the quasi-1D superconductivity in Ta_2PdS_5 nanowires, which are summarized as below:

1. The *I-V* characteristic of Ta_2PdS_5 nanowire exhibits multiple steps in transition from superconducting to the normal state, which is due to the phase-slip process in quasi-1D superconductors[8, 30]. As the thickness of the Ta_2PdS_5 nanowire goes thinner, the enhanced Ohmic finite resistance emerges due to the 1D confinement effect consistent with thermal-active phase-slip model[8, 28, 29]. The temperature-dependent critical current fits the Bardeen's formula[2, 3] for quasi-1D superconductors quite well, indicating the quasi-1D superconductivity of Ta_2PdS_5 nanowires.
2. The actual size of the nanowires samples is smaller than the penetration depth of

Ta_2PdS_5, which satisfies the condition required for quasi-1D superconductivity[1, 3].

3. The angle-dependent B_{C2} of the Ta_2PdS_5 nanowire is highly anisotropic. The angle-dependent B_{C2} of bulk Ta_2PdS_5 fits the 3D model well, while the angle-dependent B_{C2} of thin nanowires do not fit either 3D or 2D model. This indicates the quasi-1D superconductivity in Ta_2PdS_5 nanowires.
4. The unique weak coupled Ta-S chains[14, 17, 23] along *b*-axis which are responsible for the superconductivity could make the superconductivity quasi-1D[24, 25].
5. The good fitting of the $z\nu$ value to 1D equation $z\nu = C|B_C^{*} - B|^{-1.2}$, as predicted by theory[31], also suggests the quasi-1D superconductivity in Ta_2PdS_5 nanowires.

Thus, we strongly believe that the superconductivity in Ta_2PdS_5 nanowire is quasi-1D in nature.

Supplementary References

1. Ning W., *et al.* Superconductor-insulator transition in quasi-one-dimensional single-crystal Nb_2PdS_5 nanowires. *Nano Lett.* **15**, 869-875 (2015).
2. Damaschke B., Yang X. & Tidecks R. The hysteresis of the critical current in superconducting tin whiskers. *J. Low Temp. Phys.* **70**, 131-150 (1988).
3. Tidecks R. *Current-induced nonequilibrium phenomena in quasi-one-dimensional superconductors*. Springer (2006).
4. Tsen A. W., *et al.* Nature of the quantum metal in a two-dimensional crystalline superconductor. *Nat. Phys.* **12**, 208-212 (2015).
5. Xing Y., *et al.* Ising Superconductivity and Quantum Phase Transition in Macro-Size Monolayer $NbSe_2$. *Nano Lett.* **17**, 6802-6807 (2017).
6. Iwasa Y., Saito Y., Kasahara Y., Ye J. & Nojima T. Metallic ground state in an ion-gated two-dimensional superconductor. *Science* **350**, 409-413 (2015).
7. Shen S., *et al.* Observation of quantum Griffiths singularity and ferromagnetism at the superconducting $LaAlO_3/SrTiO_3(110)$ interface. *Phys. Rev. B* **94**, 144517 (2016).
8. Tian M., *et al.* Dissipation in quasi-one-dimensional superconducting single-crystal Sn nanowires. *Phys. Rev. B* **71**, 104521 (2005).
9. Xi X., *et al.* Ising pairing in superconducting $NbSe_2$ atomic layers. *Nat. Phys.*, 139-143 (2015).
10. Zeng J., *et al.* Gate-Induced Interfacial Superconductivity in 1T-$SnSe_2$. *Nano Lett.* **18**, 1410-1415 (2018).
11. Lu J. M., *et al.* Evidence for two-dimensional Ising superconductivity in gated MoS_2. *Science* **350**, 1353-1357 (2015).
12. Tinkham M. *Introduction to superconductivity*. Courier Corporation (2004).
13. Qin F., *et al.* Superconductivity in a chiral nanotube. *Nat. Commun.* **8**, 14465 (2017).

14. Khim S., *et al.* Enhanced upper critical fields in a new quasi-one-dimensional superconductor Nb_2PdxSe_5. *New J. Phys.* **15**, 123031 (2013).
15. Azevedo L., *et al.* The upper critical field of superconducting polysulfur nitride,$(SN)_x$. *Solid State Commun.* **19**, 197-201 (1976).
16. Zhang Q. R., *et al.* Anomalous metallic state and anisotropic multiband superconductivity in $Nb_3Pd_{0.7}Se_7$. *Phys. Rev. B* **88**, 024508 (2013).
17. Lu Y., *et al.* Superconductivity at 6 K and the Violation of Pauli Limit in $Ta_2Pd_xS_5$. *J. Phys. Soc. Jpn.* **83**, 023702 (2014).
18. Xu C., *et al.* Large-area high-quality 2D ultrathin Mo_2C superconducting crystals. *Nat. Mater.* **14**, 1135-1141 (2015).
19. Werner T., Tidecks R. & Johnston B. Whiskers from the In-Pb system: Growth, handling and characteristic properties. *J. Cryst. Growth* **73**, 467-481 (1985).
20. Schulz U. & Tidecks R. Dissipative state of superconducting zinc whiskers. *J. Low Temp. Phys.* **71**, 151-171 (1988).
21. Slama G. & Tidecks R. The influence of the electron mean free path on phase-slip centers in indium whiskers. *Solid State Commun.* **44**, 425-429 (1982).
22. Li P., *et al.* Switching currents limited by single phase slips in one-dimensional superconducting Al nanowires. *Phys. Rev. Lett.* **107**, 137004 (2011).
23. Zhang J., *et al.* Superconductivity at 2.5 K in the new transition-metal chalcogenide Ta_2PdSe_5. *Supercond. Sci. Technol.* **28**, 115015 (2015).
24. Scheidt E. W., Hauf C., Reiner F., Eickerling G. & Scherer W. Possible indicators for low dimensional superconductivity in the quasi-1D carbide Sc_3CoC_4. *Journal of Physics: Conference Series* **273**, 012083 (2011).
25. Bergk B., *et al.* Superconducting transitions of intrinsic arrays of weakly coupled one-dimensional superconducting chains: the case of the extreme quasi-1D superconductor $Tl_2Mo_6Se_6$. *New J. Phys.* **13**, 103018 (2011).
26. Elmurodov A. K., *et al.* Phase-slip phenomena in NbN superconducting nanowires with leads. *Phys. Rev. B* **78**, 214519 (2008).
27. Tidecks R. & Slama G. Breakdown of superconductivity in current-carrying indium whiskers. *Zeitschrift für Physik B Condensed Matter* **37**, 103-113 (1980).
28. Lukens J. E., Warburton R. J. & Webb W. W. Onset of Quantized Thermal Fluctuations in "One-Dimensional" Superconductors. *Phys. Rev. Lett.* **25**, 1180-1184 (1970).
29. Newbower R., Beasley M. & Tinkham M. Fluctuation effects on the superconducting transition of tin whisker crystals. *Phys. Rev. B* **5**, 864 (1972).
30. Michotte S., Mátéfi-Tempfli S., Piraux L., Vodolazov D. Y. & Peeters F. M. Condition for the occurrence of phase slip centers in superconducting nanowires under applied current or voltage. *Phys. Rev. B* **69**, 094512 (2004).
31. Motrunich O., Mau S.-C., Huse D. A. & Fisher D. S. Infinite-randomness quantum Ising critical fixed points. *Phys. Rev. B* **61**, 1160 (2000).
