# Supplementary material for: Signature of quantum Griffiths singularity state in a layered quasi-one-dimensional superconductor
Source: Nat Commun. 2018 Nov 7;9:4656. doi: 10.1038/s41467-018-07123-y (PMC6220168; doi:10.1038/s41467-018-07123-y)
Supplement: Supplementary file 2 — Description of Additional Supplementary Files [file 41467_2018_7123_MOESM2_ESM.pdf]

Supplementary Data legend

Source data file for Fig 4.
